# Pompion Seed as a Tæniafuge

**Published:** 1856-10

**Authors:** John W. Jones

**Affiliations:** Atlanta, Georgia


### ARTICLE III.

*Pompion Seed as a Tæniafuge. By* JOHN W. JONES, M. D., *of Atlanta, Georgia.*

As the therapeutical properties of each and all the articles that compose the Materia Medica can only be fully ascertained by repeated experiment and observation; and as it is the duty of every physician to make known to the profession any and all facts of which he may be cognizant, (however minor in character they may appear,) and especially those which lead to practical utility; and as the discovery of a remedy is not alone sufficient to establish its reputation and ensure its general employment, I am therefore induced to contribute the mite contained in the following communication:

"Having seen in the journals of several past years, authentic accounts of the successful employment of the seed of the cucurbita pepo for the expulsion of that genus of intestinal worms denominated Tænia, and being aware of the difficulty that had hitherto attended their speedy and safe removal, I at once became anxious for an opportunity to test the merits of this recently discovered remedy; but which did not present itself until the autumn of 1853. About that period, I was consulted by Mrs. D——, a very intelligent and amiable lady of Montgomery county, Alabama, who informed me that she had suffered long and much from the presence of a Tape worm. Her appearance but too plainly indicated the reality of her statements. She had no doubt of the existence of the parasite in her case, for in addition to other symptoms, she had several times discharged small portions of it, from which, however, her sufferings did not materially abate. She, together with her numerous friends, had pretty well despaired of her recovery. Many remedies had been resorted to, and some of quite a potent character. Among those she mentioned, were a protracted system of semi-stanation and hydropathy, in all its forms. Remembering the flattering accounts given of the new remedy in the then recent journals, and more particularly that from the pen of Prof. Henry S. Patterson, M. D., in which he says: "in the Medical Examiner for October 1852, I reported a case of radical cure of tænia, by the use of an emulsion of pompion seed, after Ol Terebenth and even Kousso had signally failed. Several other cases have been reported both before and since mine, all going to establish the efficacy of this new remedy. Should it prove as generally successful in expelling the worm as the cases indicate, it will become a valuable accession to our means of treatment in a troublesome and obstinate affection." I say, influenced by this and other simular reports, I advised a strong decoction of the pumpkin seed, with special directions as to the manner of its preparation and administration. The patient left me somewhat encouraged, and with a promise forthwith to try the prescription. But it was not until January thereafter that she used the remedy. When quite unexpectedly, and to the great joy of herself, husband and friends, she discharged an entire Tape worm, more than 20 feet in length, from which time she gradually improved, and is now enjoying good health.

The author just quoted, says, "the seed of the common pompion consist of a leathery white envelope, enclosing an oily albumen of a slightly greenish tinge. They are modorous and have a sweetish mucilaginous taste. Rubbed up with warm water or milk, and sweetened, they form a very pleasant emulsion; and this is the way in which they have generally been administered. They abound in fixed oil, which is readily yielded on expression, and appears to be the only constituent of any importance. Conceiving this oil to be the authelmintic principle, I determined to use it in the first case of tænia I should encounter." H.

When Prof. Patterson made this communication, he had not used this oil as a tæniafuge, as will be perceived by his remarks on this subject, but informs us that Mr. John C. Lyons, then a medical student residing near Kensington, Pennsylvania, had used it in a case with the "happiest result." Mr. Lyons gave half an ounce of the oil as a dose, and repeated it in two hours, and then followed it with castor oil.

The remedy may be prepared and used as follows: Take halt a pound of the seed well bruised, and three pints of water, and reduce the mixture by means of a gentle heat to a pint and a half. Then strain the liquid from the seed, and give a wine glassful of the former (or more if the stomach will retain it) every four or six hours, until some six draughts are taken, to be succeeded by some brisk cathartic. The patient should eat sparingly, or fast, for some twelve or twenty-four hours prior to, and while using the decoction.

The testimony that has already accumulated in favor of this remedy, warrants the conclusion that it is at least among, if not the most prompt, safe and reliable means now known for the expulsion of tænia. It is a fact long since well known to the profession generally, that the oleum terebinthina and the bark of the root of the Punica Granathum would each frequently expel the tape worm from the intestines; nevertheless, they are, in some respects, objectionable. The former, in small doses, is apt to produce severe strangury, and in larger quantities, giddiness, intoxication, &c.; and the latter article, in full doses, often gives rise to vertigo, abdominal pains and other disagreeable sensations. With the lights betore us, we would therefore recommend the cucurbita pepo in preference to the former, and to all other remedies for the expulsion of tænia. The infrequency of such cases renders it measurably impracticable for any one practitioner to make all the necessary observations upon this article, in the treatment of this distressing affection; and hence the propriety of congregating the isolated experience of many as to its merits or demerits. Whether it will prove equally efficacious in removing other varieties of worms from the alimentary canal, is a question yet to be determined.

